# MicroRNAs as Biomarkers for Myocardial Infarction

**DOI:** 10.1007/s11883-012-0238-z

**Published:** 2012-03-06

**Authors:** Kanita Salic, Leon J. De Windt

**Affiliations:** 1 Department of Cardiology, Cardiovascular Research Institute, Faculty of Health, Medicine and Life Sciences, Maastricht University, Maastricht, The Netherlands; 2 Interuniversity Cardiology Institute Netherlands, Royal Netherlands Academy of Sciences, Utrecht, The Netherlands; 3 Department of Cardiology, Cardiovascular Research Institute Maastricht, Maastricht University, Maastricht, The Netherlands

**Keywords:** Circulating microRNAs, Biomarkers, Myocardial infarction, Cardiovascular diagnostics

## Abstract

MicroRNAs (miRs) are short non-coding RNA molecules involved in post-transcriptional gene regulation by binding to the 3′ untranslated region of a messenger RNA (mRNA), thereby inhibiting the translation or inducing mRNA destabilization. MiRs are generally considered to act as intracellular mediators essential for normal cardiac function, and their deregulated expression profiles have been associated with cardiovascular diseases. Recent studies have revealed the existence of freely circulating miRs in human peripheral blood, which are present in a stable nature. This has raised the possibility that miRs may be released in the circulation and can serve as novel diagnostic markers for acute or chronic human disorders, including myocardial infarction (MI). This review summarizes the recent findings of miRs that fulfill the criteria of candidate biomarkers for MI.

## Introduction

Acute myocardial infarction (MI) is a major cause of mortality and morbidity in Western hemisphere. Annually, around 15 million patients in the United States and Europe are presented to the emergency department with chest pain or other symptoms indicative of MI [1, 2]. Early diagnosis and treatment of patients with acute MI could prevent or reduce ischemic damage to the myocardium and, therefore, could prevent subsequent cardiac remodeling and failure.

MI reflects cardiomyocyte death (necrosis) as a consequence of prolonged ischemia [3], which results from acute thrombotic occlusion of a coronary artery. Myocardial cell death can be detected by release of multiple proteins from the damaged cells into the circulation [4]. Cardiac troponin (Tn) I and T are structural proteins predominantly expressed in the heart, and are currently considered as the “gold standard” for acute MI (AMI) [4]. Their detection in peripheral blood indicates cardiomyocyte necrosis, and in combination with 12-lead electrocardiogram (ECG) and creatine kinase isoenzyme MB (CK-MB) they form the diagnostic cornerstones for MI [2, 5, 6]. However, although elevated biomarkers in the blood indicate myocardial damage, they do not diagnose the underlying mechanisms. For example, elevated values of the biomarkers in the absence of clinical evidence of ischemia could also occur as a consequence of other causes of cardiac injury, such as myocarditis, sepsis, cardioversion, or ablation [7–10]. Cardiac troponins are superior to all other biomarkers that have been clinically available in the diagnosis of MI [11–14] and they directly correlate with the size of MI [15, 16]. A major drawback of the contemporary Tn assays is their inadequate sensitivity during the first few hours after the onset of MI, as they are released slowly from damaged cardiomyocytes and do not peak until 6 to 12 h after the onset of symptoms [17]. Therefore, sampling is needed every 6 h. However, high-sensitive Tn assays are already on the market, and recent multicenter studies [18•, 19•] have shown that these assays improved the early diagnosis of MI, even if patients are presented within 3 h after the onset of chest pain. These assays contributed to safely “rule out” or “rule in” coronary causes of acute chest pain.

microRNAs (miRs) are evolutionary conserved, short, non-coding (~ 22 nucleotides) RNA molecules involved in post-transcriptional gene regulation [20–22]. Their binding potency to the 3′ untranslated region (UTR) of messenger RNAs (mRNAs) determines their mode of action: translational inhibition and/or augmented mRNA degradation, both pathways resulting in endogenous gene silencing. Around 1500 human miRs (http://www.mirbase.org/ v 18.0) have been cloned and sequenced and it is estimated that they regulate up to 50% of the protein-coding genes [23–25].

MiRs are generally considered to act as intracellular mediators essential for normal cardiac function [26] and their deregulated expression profiles have been associated with cardiovascular diseases [26–29]. Recent studies have revealed existence of freely circulating miRs in human peripheral blood that are stably expressed [30–32]. This has raised the possibility that microRNAs may be probed in the circulation and can serve as novel diagnostic markers for cardiovascular diseases. An important characteristic of miRs is their tissue and cell specificity [33] providing unique signature with diagnostic opportunities for diverse diseases [34]. In line with this, several studies have demonstrated circulating miRs reflecting pathologic condition, such as cancer and liver injury indicating that miRs could be used as sensitive and specific biomarkers of various pathologies and tissue injuries [35, 36]. In addition, another advantage is the relative ease with which miRs can be measured: polymerase chain reaction (PCR) allows being more sensitive and specific than classic antibody-based assays. A major improvement comes within reach to measure dozens of circulating miRs at once. This could become feasible against reasonable costs, comparable to the costs of measurement of only two or three protein markers. This induces a completely novel way of designing and using biomarkers in heart disease: instead of seeking one or two gold standard diagnostics, a more complete profile can be routinely measured to allow sensitive characterization of subtypes of disease. It is already known for classical biomarkers that adding a novel marker to the existing ones adds information and increases diagnostic power but also sharply increases the associated costs. Designing oligonucleotides for miRs and performing subsequent quantitative reverse transcriptase (qRT)-PCRs are much less time- and cost-consuming processes when compared to the development and production of new and specific antibodies and enzyme-linked immunosorbent assays (ELISAs). Therefore, circulating miRs as biomarkers for MI could increase the number of markers measured and continue to increase diagnostic power without increasing costs to a larger extent.

Ideal blood biomarkers of MI should comprehend the following characteristics: they should be 1) abundant and preferably exclusively expressed in the tissue of interest, 2) expressed at low levels in the blood under normal/healthy circumstances, 3) released into the circulation after tissue injury, 4) stable in the circulation, and 5) easily detected with high sensitivity and specificity. Here we will review the recent findings of circulating miRs that fulfill the mentioned criteria and discuss their potential to be used as biomarkers for MI (Table 1).

**Table 1:** Circulating microRNAs in acute myocardial infarction studies

microRNA	Fold change, increase or decrease	ROC (AUC)	Correlation	Time of blood sampling	No. samples	Species	Reference
miR-1	300↑	0.98	TnT	< 12 h, > 12 h, day 2, day 3, > 1 month	25 STEMI,	Human	[39••]
miR-133a	70↑	0.86			11 healthy	Pig	
miR-208b	3000↑	1.00			6 closed		
miR-499	250↑	0.99			chest MI		
miR-208a	nd						
miR-1	60↑			0, 30, 60, 90, 120 and 150 min			
miR-133a	60↑						
miR-208b	80↑						
miR-499	60↑						
miR-1	All four significantly ↑	0.85		4.8 ± 3.82	33 AMI,	Human	[40••]
miR-133a		0.87			17 non-CHD, 16 CHD		
miR-208a		0.97			6 non-op,		
miR-499		0.82			6 sham-op,		
miR-1	500↑			1, 3, 6, 12, and 24 h after the ligation	6 coronary artery ligation	Rat	
miR-133a	750↑						
miR-208a	850↑						
miR-499	40↑						
miR-1	ns			< 12 h	32 AMI,	Human	[44••]
miR-133a	4↑				36 controls		
miR-208b	1600↑	0.94	TnT				
miR-499	100↑	0.92	TnT				
miR-1	16↑			517 ± 309 min	33 STEMI, 17 healthy donors	Human	[38••]
miR-133a	140↑						
miR-133b	60↑						
miR-499-5p	100↑						
miR-208a	nd						
miR-1	6↑			15 min to 5 d	4–5 coronary artery ligation and sham operated	Mouse	
miR-133a	13↑						
miR-133b	5↑						
miR-499-5p	60↑						
miR-208a	↑						
miR-499	62↑		CK-MB	48 h	9 AMI, 5 UAP, 9 CHF_III, CHF_II, normal controls	Human	[49]
miR-1	61%↑	0.77	QRS widening		93 AMI, 66 controls	Human	[41••]
mir-133	ns						
miR-1	20–100↑		CK-MB	8.5 ± 3.82	31 AMI, 20 controls	Human	[42••]
	200↑		Infarct size in vivo	0, 1 h, 3 h, 6 h, 12 h, 24 h, 3 d, 7 d, 14 d, 21 d, and 28 d	8–12 LAD ligation, 8–12, sham operated	Rat	
			Cell damage in vitro				
miR-133	4.4↑	0.89		5.24 ± 1.38 h, 20 h, 7 d	51 AMI,	Human	[69]
miR-328	10.9–16.1↑	0.81			28 controls		
miR-145	2↑		Hs-TnT	3.0 ± 2.3 h, 3 d, 4 d	20 STEMI,	Human	[52•]
miR-30c	1.3 ↑		Hs-TnT		20 controls,		
miR-1291	4.5 ↓	0.91			20 internal control		
miR-663b	2 ↓	0.94					
miR-1	↑		eGFR, hs-TnT		117 UAP,	Human	[43••]
miR-133a	↑		eGFR, age,hs-TnT		131 NSTEMI,		
miR-133b	ns		hs-TnT		196 STEMI		
miR-208a	ns		-				
miR-208b	↑		age, smoking, hs-TnT				
miR-499	ns		male gender				

*AMI* acute myocardial infarction; *AUC* area under the curve; *CHD* coronary heart disease; *CHF_II* congestive heart failure, NYHA class II; *CHF_III* congestive heart failure, NYHA class III; *CK-MB* creatine kinase isoenzyme MB; *eGFR* estimated glomerular filtration rate; *hs-TnT* high-sensitive TnT; *LAD* left anterior descending artery; *MI* myocardial infarction; *miR* microRNA; *nd* not detected; *non-op* non-operated; *ns* not significant; *NSTEMI* non-ST elevation MI; *ROC* receiver operator characteristics; *sham-op* sham operated; *STEMI* ST elevation myocardial infarction; *TnI* troponin I; *TnT* troponin T; *UAP* unstable angina pectoris.

## miR-1 and miR-133

miR-1-1/miR-133a-2 and miR-1-2/miR-133a-1 are two bi-cistronic miR clusters expressed in both skeletal and cardiac muscle. The sequence of mature miR-1-1 and miR-1-2 is identical and the same holds true for miR-133a-1 and miR-133-2. MiR-133b differs from miR-133a in the 2 nucleotides at the 3′ terminus and is specifically expressed in skeletal muscle.

miR-1 is the most abundantly expressed miR in the heart. Both miR-1 and miR-133 are anti-hypertrophic miRs, involved in regulation of signaling cascades and sarcomeric organization [37]. Six hours after coronary artery occlusion in mice, miR-1 and miR-133 levels were decreased in infarcted and border zone area of the heart whereas their levels were increased in the peripheral blood [38], indicating release of these miRs into the circulation after tissue injury. Also, in AMI patients, significantly higher circulating levels of miR-1 and miR-133 levels could be detected in the circulation [38••, 39••, 40••, 41••, 42••, 43••, 44••] as soon as 5 h after the onset of symptoms [40••]. In addition, when plasma samples were obtained 156 min after the onset of symptoms, miR-1 and miR-133 levels had a significantly higher peak than TnI, indicating their early release after MI [38]. However, one study showed no significant differences in miR-1 expression between AMI and controls [44••], which could be explained by possible renal elimination of miR-1 [39••] or by conceivable presence of higher skeletal muscle turnover in the control patients [40••]. In addition, enhanced miR-1 and miR-133 levels in the circulation were detected after sham operated hearts [40••], demonstrating that damaged muscle could also contribute to induced levels of mR-1 and miR-133 in the circulation.

Both miR-1 and miR-133 levels augment in the circulation of the ST-elevated MI (STEMI) patients compared to the healthy controls within the first 12 h after the onset of the symptoms, followed by a return to adjacent baseline levels after 12 to 24 h [39••]. In contrast, one study demonstrated significantly high levels of miR-133 in the plasma after 20 h of the onset of the symptoms [35], although not precisely describing which miR-133 isoform was studied. In 8 of 25 STEMI patients, miR-1 and miR-133a could be detected in the urine within 24 h after the onset of symptoms indicating renal elimination. This is in line with another study showing a correlation between miR-1 and miR-133a and estimated glomerular filtration rate (eGFR) in STEMI patients but not between miR-133b and eGFR [43••]. Furthermore, both miR-1 and miR-133 correlated with TnT [43••] implying myocardial damage. Moreover, miR-1 also positively correlates with creatine kinase-MB [42••] and QRS widening in AMI patients [41], with infarct size in an animal model of MI and with cell damage in vitro [42••].

Receiver operator characteristics (ROC) curves for miR-1 demonstrate area under the curve (AUC) varying from 0.77 [41••] to 0.98 [39••, 40••] indicating fair to excellent accuracy of this miR for the use of a biomarker in MI. For miR-133, the ROC analysis reveal AUC values between 0.86 and 0.89 indicating good sensitivity and specificity distinguishing between STEMI patients and healthy controls [39••, 40••]. Together with their high expression in the heart, and increased levels in the blood after the MI, miR-1 and miR-133 could be suitable candidates as biomarkers for the MI. However, other injuries to the skeletal muscle should be preferably excluded to rule out the non-specific effects. In addition, the oscillations of these miRs after MI should be studied accurately to reveal their exact times of increase and decrease in the circulation and to study to which extent they could contribute to the already existing biomarkers. Finally, larger study populations are essential to make correct conclusions if miR-1 and miR-133 could be used as biomarkers in MI.

## miR-499 and miR-208

miR-499 is an evolutionary conserved muscle-specific miR that is located in an intronic region of the *MYH7B* gene and plays a role in myosin gene regulation [45, 46]. Although miR-499 is highly expressed under normal conditions in the heart [45, 47], its expression decreases in the ischemic heart [48], suggesting its release from the damaged tissue. Indeed, miR-499 levels could be detected in plasma of AMI patients [38••, 39••, 40••, 44••, 49], whereas one study did not detect differences in miR-499 expression between STEMI, non–ST-elevated MI (NSTEMI), and patients with unstable angina [43••]. Although miR-1 and miR-133a peaked around 156 min after the onset of MI symptoms, miR-499 is at its highest point around 12 h [38••, 39••], suggesting possible slower kinetics compared to miR-1 and miR-133. However, similar to miR-1 and miR-133, miR-499 levels increase in the circulation in the sham operated hearts, indicating that damaged muscle contributes to enhanced circulating miR-499 levels.

Besides parallel expression of miR-499 and TnI in an animal model of MI [38••], miR-499 correlates with TnT [44••] and CK-MB [49] in AMI patients. ROC analysis demonstrates AUC values varying between 0.82 and 0.99, indicating good to excellent sensitivity and specificity [39••, 40••, 44••].

Other miRs encoded by the myosin genes are miR-208a and miR-208b, which are located in *MYH6* and *MYH7* genes, respectively. MiR-208a and miR-208b are abundantly and exclusively expressed in the heart, making them most suitable candidates to be used as biomarkers for the MI. Indeed, miR-208a [40••] and miR-208b [39••, 43••, 44••] levels are increased in the circulation after the MI. Two studies showed that miR-208b was the most abundantly elevated miR in the plasma, with 1600 [44••] and 3000 [39••] times more expression in the AMI patients than in the controls. Furthermore, miR-208b levels correlated with TnT levels reflecting myocardial damage [39••, 43••, 44••]. Moreover, the amount of miR-208b in blood inversely correlates with left ventricular ejection fraction, raising the possibility in using this miR not only for diagnostic purposes but also for potential prognostic use in long-term cardiac function and probability of developing heart failure. Nevertheless, one larger population study of 444 subjects revealed that although circulating levels of miR-208b were associated with all-cause mortality at 6 months, after adjustment for TnT levels, its association was lost with the outcome. This implies that miR-208b does not add prognostic information to an already sensitive necrosis marker. However, it should be taken into account that this does not exclude the potential of miR-208b to augment its prognostic value when measuring at an earlier or later time point.

Only one study detected significantly higher circulating miR-208a levels in the AMI patients [40••] in contrast to the others [38••, 39••, 49]. This could have several explanations. The first is related to the time point of sampling and could explain why miR-208a was not detected in the circulation [49]. In this study, blood sampling occurred within 48 h after the onset of symptoms [49], although miR-208a peaks around 3 h after the AMI [38••, 40••] and is restored to the baseline after 24 h [40••]. The second explanation is related to the myosin isoform expression between a mouse and a human heart. miR-208a is abundantly and exclusively expressed in the adult mouse heart, whereas miR-208b is more expressed during development and in response to stress. However, *MYH7*, harboring miR-208b, is the primary isoform in the human cardiac muscle [50, 51], providing an explanation why miR-208b is abundantly present in the human heart. This could explain why two studies could not detect miR-208a in the circulation, although sampling the blood within the appropriate time line [38••, 39••].

Specificity and sensitivity analysis for miR-208b reveal AUCs between 0.94 and 1.00, indicating excellent accuracy for discrimination between AMI patients and the controls [39••, 44••]. In combination with its exclusive expression in the heart, and high increase in blood after the MI, miR-208b could be a suitable candidate as a biomarker for the MI. Nevertheless, the kinetics of miR-208a should be studied extensively and in bigger populations to reveal its exact incline and decline after the MI and to decide what its contribution could be to the already existing biomarkers such as hs-TnT and TnI.

## Non-specific Cardiac and Skeletal Muscle miRs

In theory, candidate biomarkers for MI should display cardiac specific expression patterns. Recently, one study revealed miR-663b and miR-1291 as the most predictive miRs for the MI with 92.5% and 85% accuracy, respectively [52•]. In addition, miR-30c and smooth muscle cell-enriched miR-145 were significantly increased in these STEMI patients and correlated with hs-TnT, indicting their potency to reflect cell death. Moreover, the same study reported an miRs signature, which could serve as a new class of biomarkers [52•]. This signature represents a combination of 20 most up- and down-regulated miRs in 20 STEMI patients in contrast to the controls and could enhance diagnostic discrimination of STEMI patients from the control with 96% specificity, 90% sensitivity, and the AUC of 0.99, indicating excellent accuracy. These results are promising, but because these miRs are not cardiac specific, other pathologic processes could underlie the effects seen in this study. Furthermore, the patient population is too small to drawn correct conclusions about this particular miRs signature and because these miRs are isolated from the whole peripheral blood, circulating cells could also contribute to the miRs expression levels. Nevertheless, such a multi-marker approach could be of interest to increase diagnostic power and to gain more information about the time line of MI, which in turn could lead to faster and accurate treatments and prevent subsequent cardiac remodeling and failure.

## Stability of Circulating miRs

Extracellular miRs have been found recently in multiple human body fluids, such as blood plasma, urine, and saliva [30, 53, 54]. MiRs are surprisingly stable in the plasma regardless of high RNAase activity [30], contributing to the possibility that that miRs could be used as new class of blood-based biomarkers. Several mechanisms influencing the miR stability in the circulation are discussed below.

Circulating miRs can be carried in different types of vesicles, such as exosomes [55•] and microvesicles [56•]. Exosomes are small (50–90 nm) vesicles that are released into the extracellular environment by fusion of the multivesicular bodies and the plasma membrane. Production of exosomes occurs through the inward budding of the endosomes. One study showed that 121 miRs are associated with exosomes and that several miRs were abundantly expressed in exosomes compared to the cells, suggesting packing regulation [55•]. These miRs are not localized on the external structures or macromolecules, but instead are restricted to the inner part [55•]. Besides exosomes, microvesicles can also contain miRs [56•]. These particles are larger (up to 1um) and shed from the cell membrane. In addition, microvesicles are enriched in bio-active molecules and contain nucleic acids and/or proteins [57]. MiRs located in exosomes or microvesicles are extremely resistant to RNAase-dependent degradation with necessity to first destroy the lipid bilayer of the vesicles before miRs can become accessible for RNAase degradation [55•].

Previous studies have shown that nucleic acids can bind and form stable complexes with specific lipids found on lipoproteins [58–60] by divalent cation bridging [61]. High-density lipoproteins (HDL) are used as carrier to deliver lipophilic anti-tumor drug to human hepatocellular carcinoma cells in vitro [62]. In animal models, liposomes containing apolipoprotein A-I, which is the main protein component of HDL, have been used to the delivery of several siRNAs to the liver [63]. Recently, one study revealed that HDL (8–12 nm) isolated from patients with hypercholesterolemia contained small RNAs including miRs [64•]. The exact mechanism of how miRs are loaded into the HDL particles and how they are protected from the external RNAases has to be elucidated.

miRs can also be stabilized in the circulation when localized in the apoptotic bodies. One study demonstrated that endothelial cell-derived apoptotic bodies, which are produced during atherosclerosis, carry miR-126 as well as other miRs [65]. The majority of extracellular miRs in blood plasma are bound to protein complexes protecting these miRs from degradation. In line with this, one study has shown that a RNA binding protein, nucleophosmin 1 (NPM1), and nucleolin were highly abundant in the fibroblast medium after serum deprivation and that especially NPM1 could bind and protect miR-122 against degradation [66]. However, it needs to be investigated if NPM1 is present and operative in binding and protecting miRs in the human circulation.

Very recently, two studies have demonstrated that vesicle-encapsuled miRs represent only a minor fragment of circulating miRs and that over 90% of circulating miRs are exosome free and bound to Argonaute proteins [67•, 68]. These proteins are naturally occurring within the cell and are a part of RNA-induced silencing complex. Argonaute 2 (Ago2) plays an especially important role in stabilizing miRs in the plasma as Ago2/miRs complexes are extremely nuclease and protease resistant.

In conclusion, several mechanisms are described for miRs export and their subsequent stabilization in the circulation. Export of most miRs is energy dependent and active transport, suggesting complex regulation of this process [66]. However, miR release after an AMI would reflect a rather passive form due to necrosis. Mature miRs in the cells that are Ago2 bound and necessary for the stabilization are localized in the cytoplasm or in the P-bodies, suggesting that extracellular miRs detected in the plasma after an AMI would represent Ago2-bound miRs.

## Conclusions

Myocardial-derived miRs, such as miR-1, miR-133, miR-499, and miR-208, might be useful as potential biomarkers for MI. These miRs are abundantly expressed in the heart but less so in the circulation under normal/healthy circumstances. In addition, they are released into the circulation after the MI, where they are in a stable conformation and can be detected with high accuracy. Larger study populations and exact time points of blood sampling are required to study the kinetics of these myocardial-derived miRs in AMI patients and to investigate their potential to be used as new biomarkers in MI. It is already known for classical biomarkers that adding a novel marker to the existing ones adds information and increases diagnostic power but also sharply increases the associated costs. Designing primers for miRs and performing subsequent qRT-PCRs are much less time- and cost-consuming processes when compared to the development and production of new and specific antibodies and ELISAs. In this way, a multi-miRs marker approach also becomes attractive, which could lead to increased diagnostic power and provide more information about the time line of MI, with the faster and accurate treatments with the subsequent improvement of the clinical outcome. To promote the use of miRs as biomarkers, it is essential to develop new techniques that can provide quick detection of miRs in the circulation.
